# Implementation of human factors engineering approach to improve environmental cleaning and disinfection in a medical center

**DOI:** 10.1186/s13756-020-0677-1

**Published:** 2020-01-16

**Authors:** I-Chen Hung, Hao-Yuan Chang, Aristine Cheng, Mei-Wen Chen, An-Chi Chen, Ling Ting, Yeur-Hur Lai, Jann-Tay Wang, Yee-Chun Chen, Wang-Huei Sheng

**Affiliations:** 1 Center for Infection Control, National Taiwan University Hospital, No.7, Zhongshan S. Rd, Taipei City, 100 Taiwan; 2 School of Nursing, National Taiwan University, No.1, Sec. 1, Jen Ai Rd, Taipei City, 100 Taiwan; 3 Department of Nursing, National Taiwan University Hospital, Taipei, Taiwan; 4 Department of Internal Medicine, National Taiwan University Hospital, Taipei, Taiwan; 5 Department of Nursing, National Taiwan University Cancer Center, No.57, Ln. 155, Sec. 3, Keelung Rd, Taipei City, 106 Taiwan; 6 Department of Medical Education, National Taiwan University Hospital, No.7, Zhongshan S. Rd, Taipei City, 100 Taiwan

**Keywords:** Environmental cleaning and disinfection, Human factors engineering, Fluorescent marker, ATP bioluminescence assay, Carriage incidence of multidrug-resistant organism

## Abstract

**Background:**

Inadequate hospital cleaning may contribute to cross-transmission of pathogens. It is important to implement effective cleaning for the safe hospital environment. We conducted a three-phase study using human factors engineering (HFE) approach to enhance environmental cleanliness.

**Methods:**

This study was conducted using a prospective interventional trial, and 28 (33.3%) of 84 wards in a medical center were sampled. The three-phases included pre-intervention analysis (Phase 1), implementing interventions by HFE principles (Phase 2), and programmatic analysis (Phase 3). The evaluations of terminal cleaning and disinfection were performed using the fluorescent marker, the adenosine triphosphate bioluminescence assay, and the aerobic colony count method simultaneously in all phases. Effective terminal cleaning and disinfection was qualified with the aggregate outcome of the same 10 high-touch surfaces per room. A score for each high-touch surface was recorded, with 0 denoting a fail and 10 denoting a pass by the benchmark of the evaluation method, and the total terminal cleaning and disinfection score (TCD score) was a score out of 100.

**Results:**

In each phase, 840 high-touch surfaces were collected from 84 rooms after terminal cleaning and disinfection. After the interventions, the TCD score by the three evaluation methods all showed significant improved. The carriage incidence of multidrug-resistant organism (MDRO) decreased significantly from 4.1 per 1000 patient-days to 3.6 per 1000 patient-days (*P* = .03).

**Conclusion:**

The HFE approach can improve the thoroughness and the effectiveness of terminal cleaning and disinfection, and resulted in a reduction of patient carriage of MDRO at hospitals. Larger studies are necessary to establish whether such efforts of cleanliness can reduce the incidence of healthcare-associated infection.

## Background

Multidrug-resistant organisms (MDRO) and *Clostridium difficile* are common causes of healthcare-associated infections (HAI) at hospitals [1]. The contaminated environment is a well-established source for transmission of these organisms [2]. Inadequate room cleaning at wards may increase the risk of acquisition of pathogens for the subsequent occupant. During a 14-month study performed at two intensive care units (ICUs), patients admitted to rooms where the prior patients carried vancomycin-resistant enterococci (VRE) had documented increased risks of VRE acquisition (hazard ratio, 4.4) [3]. A case-control study showed that cases who had been exposed to a prior infected or colonized bed occupant had a 5.83-fold increased risk in developing a HAI with the same organism [4]. Thus, it is important to implement effective terminal cleaning and disinfection at wards to prevent transmission of MDRO.

There are many options for improving environmental cleaning and disinfection, including newer disinfectants, wipes, and automated room-disinfection devices (eg, ultraviolet-C light, hydrogen peroxide vapor) [5, 6]. These interventions may incur additional costs and the effects are controversial. A study found that 26.6% of rooms remained contaminated with *Acinetobacter baumannii* complex or methicillin-resistant *Staphylococcus aureus* (MRSA) following 4 rounds of bleach disinfection [7]. The reasons for residual pathogens after terminal cleaning and disinfection might be due to incomplete wipe-off or inadequate concentration or contact time of the bleach.

Manual cleaning is a labor-intensive and repetitive task that can become monotonous. There is a need to implement effective and sustainable environmental cleaning and disinfection strategies for environmental service workers (ESWs) to remain thorough and use the right technique and product [8]. Strategy guided by human factors engineering (HFE) principles may be helpful for improving patient room cleaning and disinfection [9]. The elements of HFE include systems initiative, design-driven innovation, and improving system performance and human well-being.

Therefore, we conducted a prospective three-phase intervention study to evaluate and improve the adequacy of terminal cleaning and disinfection practices. We hypothesized that using a HFE approach to ensure consistency of wipe-off of high-touch surfaces may enhance environmental cleanliness.

## Methods

This study was conducted using a prospective and interventional trial at medical wards, surgical wards, and ICUs in a 2629-bed academic medical center. In total, 28 (33.3%) of all 84 wards were recruited by stratified-random sampling. Three-phase approach was implemented. Each phase had three consecutive months. This study was approved by the institutional review boards of the study facilities, and waivers of informed consent were granted (IRB No. 201601083RIND).

### Three-phase approach

Phase 1 (July 2016 to September 2016) served as a baseline phase, during which there were no additional interventions. At this medical center, routine disinfection of the surfaces in specific areas close to patients, such as bed rails and overbed tables, are disinfected daily in the ICUs and weekly in the general wards. Areas distant from patients, only light switches, door knobs and bathrooms are disinfected daily (targeted disinfection) in all types of wards. The disinfectant is 600 ppm sodium hypochlorite that diluted by the ESWs according to the manufacturer’s instructions (Bleach, Yuxiang Tech. Inc., Hsinchu County, Taiwan) for routine disinfection and terminal cleaning/ disinfection.

For each ward, the following 10 high-touch surfaces were tested after terminal cleaning and disinfection once per month during this phase: room light switches, room door knobs, chairs, bedside table handles, nursing calls, intravenous (IV) poles, bed rails controllers, attendant control panels, overbed tables, and bathroom door knobs in the general ward; nursing carts, wardrobe knobs, respiratory ventilator controllers, IV pump panels, suction controllers, vital signs monitor panels, electrocardiogram lead machines, bed rails controllers, attendant control panels, and overbed tables in the ICU. The evaluations were performed using a fluorescent marker (GlitterBug®, Brevis Corp., Salt Lake City, UT, USA) method [5, 10] to indicate physical cleaning actions by ESWs and using the adenosine triphosphate (ATP) bioluminescence assay (3 M Clean-Trace System; 3 M, St. Paul, MN, USA) and aerobic colony counts (ACCs) method to evaluate the effectiveness of terminal cleaning and disinfection [11, 12]. The quantitative concentration of sodium hypochlorite before use in terminal cleaning and disinfection was tested using commercial bleach meter (Mizu, Senno Tech. Inc., Taiwan) according to the manufacturer’s instructions. All samplings were performed by the same well-trained infection control nurse without announcement. ESWs were blinded to the cleaning assessment in this investigation.

After patients were discharged, the same 10 high-touch surfaces were tagged with fluorescent marker prior to terminal cleaning and disinfection. Allowing 10 min after terminal cleaning and disinfection for the surfaces to dry to avoid that residuals of the disinfectant, each high-touch surface was examined under ultraviolet light. Each high-touch surface with the same 100 cm^2^ area adjacent fluorescent marker was entirely swabbed using a close zigzag pattern by simultaneously a swab of ATP assay and a pre-moistened sterile culture swab. The ATP swab was activated in accordance with the manufacturer’s instructions, and the reading (in relative light units, RLU) was recorded. For ACC method, each culture swab was suspended in 1 mL sterile saline then vortexed for 10 s, and 0.2 mL was spread on to a tryptic soy agar with neutralizers (Creative Microbiologicals, Taipei County, Taiwan). After 48 h of incubation at 35 °C, the total numbers of colonies on the agar were calculated. For fluorescent marker, a surface with less than one quarter circle of residual gel was considered clean according to our previous approach [10]. The benchmark clean criteria of ATP assay were < 250 RLU in ICUs and < 500 RLU in general wards [11]. The benchmark clean criterion of ACC method was < 100 colony forming units (CFU)/100 cm^2^ [12]. Effective terminal cleaning and disinfection was qualified with the aggregate outcome of the same 10 high-touch surfaces in each room by the fluorescent marker, the ATP assay, and the ACC method respectively. A score for each high-touch surface was recorded, with 0 denoting a fail and 10 denoting a pass by the benchmark of the evaluation method. The full terminal cleaning and disinfection score (TCD score) was calculated out of 100.

Phase 2 (March 2017 to May 2017) was implementing environmental cleaning and disinfection strategies. The intervention began with a meeting with the infection control nurses, the resourcing supervisors of the ESWs, and the hospital administrators. Environmental cleaning and disinfection strategies were designed by HFE principles [9], including ESWs education, the redesigned workflow of terminal cleaning and disinfection, a regular method of bleach dilution, and a checklist-form reminder (Table 1). The bleach was diluted with cold water using the uniform containers by the ESWs for standardization (Additional file 1). The reminder was a form that had 14 high-touch surfaces photographs at a patient unit and had to be checked by the ESW at the first terminal cleaning and disinfection opportunity at every work day (Additional file 2). The thoroughness of terminal cleaning and disinfection practices during this phase were evaluated by three methods that were same with the sampling method during phase 1. ESWs received individual educational feedback on the outcome of each fluorescent marker audit during this phase.

**Table 1:** Human Factors Engineering (HEF)-Informed Environmental Cleaning and Disinfection Strategies for Improving Terminal Cleaning and Disinfection

Work system elements	HFE-informed intervention
People	Environmental service workers (ESWs) education (eg, strengthening wipe off practices, when to change cleaning cloths and mop heads, and reminders not to miss the high-touch surfaces.)
Tasks	Redesign and simplify the workflow of terminal cleaning and disinfection on the basis of ergonomics principles (eg, clean one side of mattress then walk to the other side to clean the other part of mattress, avoid excessive extension of body)
Tools and technologies	Redesign the regular method of disinfectant dilution (Additional file 1). Design a checklist-form reminder with high-touch surfaces photographs (Additional file 2).
Organization Internal environment External environment	Using a media to publicize the efforts of the ESWs in infection prevention, encourage all of people to maintain a clean environment in the hospital, and make understanding of time allowed to clean room.

Phase 3 (July 2017 to September 2017) was programmatic analysis and overall feedback. The phase 2 interventions were maintained and the ESWs received individual feedback of each fluorescent marker audit continuously. In this phase, the evaluation of the thoroughness of terminal cleaning and disinfection was performed again, as during phase 1 and phase 2, and overall feedback to all staffs were performed periodically.

### Outcomes

The primary outcome measure was the incidence of HAI by specific MDROs, including VRE, MRSA, and carbapenem-resistant *Acinetobacter baumannii* complex (CRABC) by the Centers for Disease Control and Prevention surveillance definitions. Secondary outcomes were the thoroughness of terminal cleaning and disinfection, including the TCD score of the fluorescent marker, the ATP assay, and the ACC method. The carriage incidence of MDRO by clinical culture results was calculated in the pre-intervention period (July 2016 to January 2017) and during the intervention period (March 2017 to September 2017). The maximum number of episodes per patient with the same MDRO in the same ward was one, even if multiple isolates of the same MDRO were cultured.

### Statistical analysis

Data were analyzed using SAS 9.4 (SAS Institute Inc., Cary, NC, USA) for descriptive statistics, paired *t* tests and normal theory tests. Repeated measures ANOVA (RM-ANOVA) was used for examining the changes of TCD scores among three phases, while ANOVA was used for comparing TCD scores among three ward types in each phase. RM-ANOVA and ANOVA with Bonferroni post-hoc tests were performed by the Statistical Product and Services Solutions version 21.0 (IBM, IBM SPSS Statistics for Windows, Armonk, NY, USA). Significant level was set as .05.

## Results

**Fig. 1:**
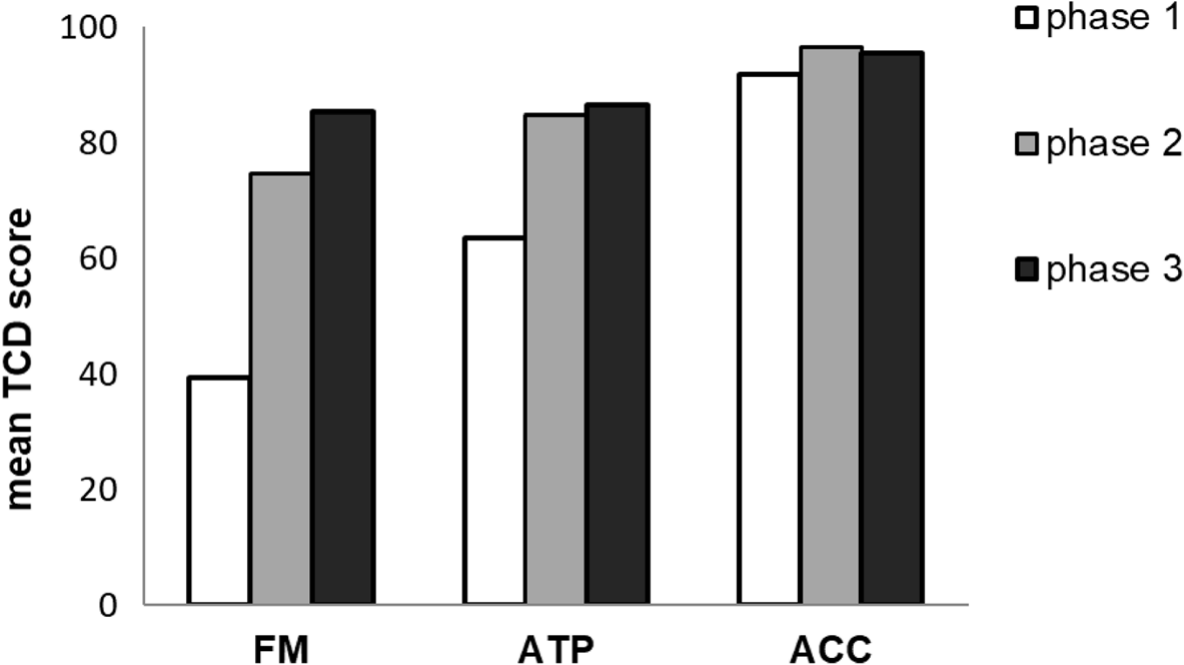
Change in the mean terminal cleaning and disinfection (TCD) score of the fluorescent marker (FM), ATP bioluminescence assay (ATP), and aerobic colony count (ACC) evaluation methods in the pre-intervention (phase 1), intervention (phase 2), and analytical phases (phase 3)

In each phase, 840 high-touch surfaces were collected from 84 rooms after terminal cleaning and disinfection. In phase 1, data from one room was missed due to emergency admission, only 830 high-touch surfaces were included in the data analysis. After the interventions, the TCD score of the fluorescent marker, the ATP assay, and the ACC method were all improved significantly (Fig. 1). The TCD scores of the fluorescent marker increased significantly from 39.4 ± 17.6, 74.6 ± 17.6, and 85.4 ± 10.0 in the pre-intervention, intervention, and analytical phases, respectively (*P* < .001). Similarly, the TCD scores using the ATP assay were 63.5 ± 13.4, 84.8 ± 11.1, and 86.6 ± 9.6 showing significant increases from phase 1 to phase 3 (*P* < .001). The TCD scores by the ACC method were also concordant 91.7 ± 6.3, 96.5 ± 4.0, and 95.6 ± 5.1 in phase 1, 2, 3, respectively (*P* = .002) (Table 2).

**Table 2:** Terminal Cleaning and Disinfection Score of the Fluorescent marker, ATP, and ACC Methods by Phases (*n* = 84)

Method	Phase 1(a)	Phase 2(b)	Phase 3(c)	*P*	Post-hoc (Bonferroni)^a^
	Mean ± SD				
Fluorescent marker	39.4 ± 17.6	74.6 ± 17.6	85.4 ± 10.0	< .001	b > a***, c > a***, c > b***
ATP	63.5 ± 13.4	84.8 ± 11.1	86.6 ± 9.6	< .001	b > a***, c > a***
ACC	91.7 ± 6.3	96.5 ± 4.0	95.6 ± 5.1	.002	b > a**, c > a*

NOTE. *ATP* ATP bioluminescence assay, *ACC* aerobic colony count, *SD* standard deviation

^a^*P < .05, **P < .01, ***P < .001

The detailed results of the evaluations by ward type was shown in Table 3. In phase 1, the lowest TCD score was 23.0 ± 13.3 by fluorescent marker among the surgical ward group. After the interventions, the TCD score of the ATP assay in the ICU group was significant lower than the TCD score of both general medical and surgical wards. After using the uniform container for disinfectant dilution, the pass rate of 600 ppm sodium hypochlorite increased incrementally from 69.9, 95.2 to 100% by phase (*P* < .001).

**Table 3:** Terminal Cleaning and Disinfection Score of the Fluorescent marker, ATP, and ACC Methods by Ward Types

Method	Medical wards (a) *n* = 30	Surgical wards (b) *n* = 27	ICUs (c) *n* = 27	*P*	Post-hoc (Bonferroni)^a^
	Mean ± SD				
Fluorescent marker					
Phase 1	39.0 ± 10.7	23.0 ± 13.3	56.5 ± 10.5	< .001	a > b*, c > a**, c > b***
Phase 2	74.7 ± 16.8	68.9 ± 21.6	80.4 ± 13.6	.339	–
Phase 3	87.3 ± 8.9	80.4 ± 12.9	88.1 ± 6.7	.196	–
ATP					
Phase 1	68.0 ± 7.2	57.0 ± 12.7	64.8 ± 17.6	.195	–
Phase 2	89.3 ± 6.4	87.4 ± 11.9	77.0 ± 11.2	.031	a > c*
Phase 3	88.0 ± 5.0	93.0 ± 6.3	78.5 ± 11.0	.002	b > c*
ACC					
Phase 1	90.3 ± 6.6	88.9 ± 6.1	96.1 ± 3.9	.027	c > b*
Phase 2	93.7 ± 4.6	98.1 ± 2.6	98.0 ± 2.6	.015	b > a*, c > a*
Phase 3	95.3 ± 3.6	96.3 ± 4.5	95.2 ± 7.1	.885	–

NOTE. *ATP* ATP bioluminescence assay, *ACC* aerobic colony count, *ICUs* intensive care units, *SD* standard deviation

^a^*P < .05, **P < .01, ***P < .001

Table 4 shows that the carriage incidence of MDRO significantly decreased from 4.1 per 1000 patient-days in the pre-intervention period to 3.6 per 1000 patient-days during the intervention period (*P* = .03). There was no changes in the incidence of HAI with the MDRO in the pre-intervention period (0.3 per 1000 patient-days) and during the intervention period (0.4 per 1000 patient-days) (*P* = .23).

**Table 4:** The Incidence of Carriage and Healthcare-Associated Infection of MDRO per 1000 Patient-Days were Determined in the Pre-intervention Period and During the Intervention Period

		The incidence of MDRO per 1000 patient-days		
	*n*	Pre-intervention period^a^	Intervention period^b^	*P*
Carriage				
Overall	28	4.1 (564/136794)	3.6 (507/140860)	.03
VRE		1.8 (253/136794)	1.3 (184/140860)	< .001
MRSA		1.3 (178/136794)	1.2 (171/140860)	.55
CRABC		1.0 (133/136794)	1.1 (152/140860)	.41
General wards	19	3.7 (421/114141)	3.4 (403/117894)	.29
VRE		1.8 (209/114141)	1.3 (152/117894)	< .001
MRSA		1.2 (136/114141)	1.2 (147/117894)	.75
CRABC		0.7 (76/114141)	0.9 (104/117894)	.07
ICUs	9	6.3 (143/22653)	4.5 (104/22966)	.01
VRE		1.9 (44/22653)	1.4 (32/22966)	.19
MRSA		1.9 (42/22653)	1.0 (24/22966)	.03
CRABC		2.5 (57/22653)	2.1 (48/22966)	.39
Healthcare-associated infection				
Overall	28	0.3 (47/136794)	0.4 (62/140860)	.23
General wards	19	0.3 (30/114141)	0.4 (44/117894)	.17
ICUs	9	0.8 (17/22653)	0.8 (18/22966)	.97

NOTE. *MDRO* multiple-drug resistant organism, including vancomycin-resistant enterococci (*VRE*), methicillin-resistant *Staphylococcus aureus* (*MRSA*), and carbapenem-resistant *Acinetobacter baumannii* complex (*CRABC*)

^a^July 2016 to January 2017

^b^March 2017 to September 2017

## Discussion

Our findings indicated that using an HFE approach to strengthen the reliable wipe offs on high-touch surfaces can significantly increase the thoroughness of the terminal cleaning and disinfection and decrease the carriage incidence of MDRO.

The use of a uniform container for disinfectant dilution and a checklist-form reminder reduced potential errors or omissions in the terminal cleaning and disinfection. These interventions improved the accuracy of disinfectant dilution and overall TCD scores. Bernstein et al. surveyed the workflow challenges of 327 ESWs at 5 hospitals, whereby only 37% ESWs reported that it was always clear what items they were responsible for cleaning, and 20% ESWs did not have enough time to perform terminal cleaning [13]. There is a high turnover rate among the outsourced ESWs, they need to be trained to understand how to do cleaning practice well and realize why their work is important, and to be recognized and certified to improve compliance and motivation [8]. Thus, the environmental cleaning and disinfection strategies are not only improving the cleaning knowledge and skills of the ESWs, but also reducing and simplifying the workflow barriers that can balance timeliness of completing tasks and optimize the cleaning practice [14]. After phase 3 of this study, we used a survey to assess knowledge and practices of environmental cleaning among the ESWs, there were 97% ESWs (65/67) agreed that the checklist-form reminder was helpful to perform terminal cleaning and disinfection well.

Studies on the different methods of cleanliness evaluation from the same high-touch surfaces demonstrated that the fluorescent marker and the ATP assay had more variations than the ACC method to define a surface as clean [10, 15]. Compatible with these studies, our data showed that the fluorescent marker had higher failure rates and lower TCD scores and the ACC method had higher pass rates and correspondingly higher TCD scores in phase 1. Although the same 10 high-touch surfaces were tested at the same time, the aggregate outcome of these evaluation methods were markedly different. Nonetheless, our data showed that the aggregate outcome of the terminal cleaning by the three different evaluation methods were all improved significantly after implementing cleaning and disinfection strategies. Like other studies [11, 16–18], the fluorescent marker and the ATP assay were useful methods for measuring the improving degree of cleanliness and could reflect more dynamic changes.

Our finding showed that the TCD score of the three evaluations in the surgical wards group were lowest in phase 1, especially the TCD score of the fluorescent marker that indicated the physical cleaning actions by ESWs. The reasons for the poor cleaning may be related to the higher turnover rates, ESW understaffing or over-load in the surgical wards [19]. We had recorded that the average number of terminal cleaning and disinfection daily by one ESW were 5.7 to 6.3 beds in the surgical wards group, 3.4 to 3.9 beds in the medical wards group, and 2.4 to 3.0 beds in the ICUs group. The overall daily hospital bed occupancy rate was approximately 92% during the study period. In the busy surgical wards, if the surface is not visibly dirty, it may not receive attention or wiping. It is important to simplify the cleaning workflow and strengthen wiping of the high-touch surfaces in a timely manner.

The outcome of the HEF is focusing on both system performance and human wellbeing [9]. Terminal cleaning and disinfection is performed at patient discharge to ensure that the patient zone is disinfected and safe for the next occupant. Our data showed that the effectiveness of terminal cleaning and disinfection was significant improved, and a significant 12.2% reduction in the MDRO carriage incidence. But the incidence of HAI with the MDRO were not change. The reason was possibly because of under-powering due to the low incidence of HAI overall, or the intervention that focus on terminal cleaning and disinfection may be insufficient to reduce the incidence of HAI with the MDRO [20, 21]. We showed that there was a significant (27.8%) reduction of VRE carriage rate, that was similar to other studies [22–24]. Datta et al. [22] reported the acquisition of both MRSA and VRE decreased significantly during the intervention periods that consisted of feedback using the fluorescent marker and bucket wetting the cleaning cloths with disinfectant; Hayden et al. [23] reported a significant 49.7% reduction of VRE acquisition rate during a period of educational intervention; Grabsch et al. [24] reported significant 24.8% reductions in newly recognized VRE acquisition and significant 66.4% reduction in environmental contamination after implementation of a cleaning-disinfection program. The impressive reductions in VRE carriage rates may be due to the unusual long-lived persistence of VRE in the hospital and human environment. VRE contamination is particularly a problem when single rooms are limited and cohorting of patients with VRE are practiced, even though contact precautions were performed for the patients colonized or infected with MDROs.

Our study has limitations. First, there were sampling-area limitations. The sampling area of the ATP assay cannot overlap the fluorescent marker to avoid overestimation of ATP values by residual fluorescence. A small surface was not clean by the fluorescent marker criterion, which did not mean that the adjacent 100-cm^2^ area had not been wiped [10]. So the data showed the TCD score of the fluorescent marker was lower than those of the other two methods. Second, we used two clean pass criteria of the ATP assay (stricter criteria in ICUs group) [11]. That was the reason the TCD score of the ATP assay in ICUs group was lower than the TCD score in general wards group after the interventions. Nonetheless, these evaluation methods of cleaning could dynamically reflect changes in the three phases. Third, we evaluated the outcome during implementing the interventions for 7 months. We were unable to verify the impact of our study on the HAI with the MDRO. The improvements of terminal cleaning and disinfection could be impacted by the Hawthorne effect of immediate feedback using the fluorescent marker. Although we did not measure other potential confounders, there were no new infection control initiatives during the study period. The hand hygiene compliance rate were 82.9% (213/257) in the pre-intervention period and 87.0% (160/184) during the intervention period at the study wards. Thus, further studies are necessary to evaluate the sustainability of such systematic improvements.

## Conclusion

Our investigations support the HFE approach to strengthen the reliability of wipe-off of high-touch surfaces and the effectiveness of terminal cleaning and disinfection. After terminal cleaning and disinfection, the hospital environment had low microbiological counts, the use of the fluorescent marker and the ATP assay may provide additional information of cleaning effectiveness. The HFE intervention resulted in a reduction in the carriage incidence of MDRO at hospitals. Larger studies are necessary to establish whether such efforts of cleanliness are effective in reducing the incidence of HAI.

## Supplementary information

**Additional file 1.** The regular method of bleach dilution. The bleach was diluted with cold water using the uniform containers by the environmental service workers.

**Additional file 2.** The checklist-form reminder of environmental cleaning.

## Data Availability

Requests for more detailed information regarding the collected data can be addressed to the corresponding author.

## Abbreviations

ACCs: Aerobic colony counts
ATP: Adenosine triphosphate
CFU: Colony forming units
CRABC: Carbapenem-resistant *Acinetobacter baumannii* complex
ESWs: Environmental service workers
HAI: Healthcare-associated infections
HFE: Human factors engineering
ICUs: Intensive care units
MDRO: Multidrug-resistant organisms
MRSA: Methicillin-resistant *Staphylococcus aureus*
RLU: Relative light units
TCD: Terminal cleaning and disinfection
VRE: Vancomycin-resistant enterococci
