# Supplementary material for: Implementation of human factors engineering approach to improve environmental cleaning and disinfection in a medical center
Source: Antimicrob Resist Infect Control. 2020 Jan 16;9:17. doi: 10.1186/s13756-020-0677-1 (PMC6966902; doi:10.1186/s13756-020-0677-1)
Supplement: Supplementary file 1 — Additional file 1. The regular method of bleach dilution. The bleach was diluted with cold water using the uniform containers by the environmental service workers. [file 13756_2020_677_MOESM1_ESM.pdf]

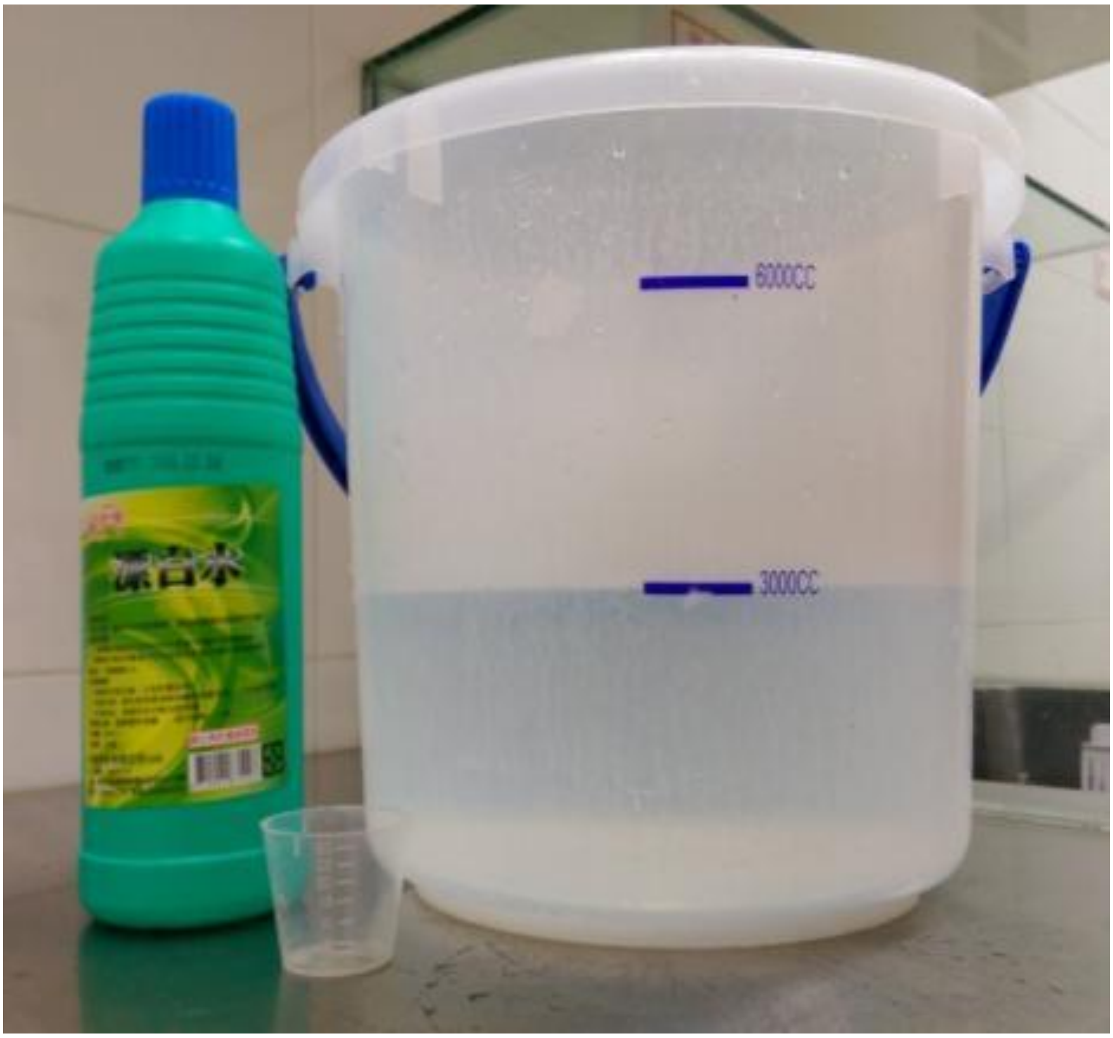

Additional file 1. The regular method of bleach dilution. The bleach was diluted with cold water using the uniform containers by the environmental service workers.
