# Supplementary material for: Implementation of human factors engineering approach to improve environmental cleaning and disinfection in a medical center
Source: Antimicrob Resist Infect Control. 2020 Jan 16;9:17. doi: 10.1186/s13756-020-0677-1 (PMC6966902; doi:10.1186/s13756-020-0677-1)
Supplement: Supplementary file 2 — Additional file 2. The checklist-form reminder of environmental cleaning. [file 13756_2020_677_MOESM2_ESM.pdf]

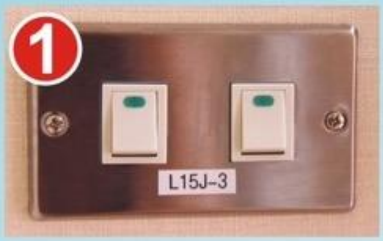

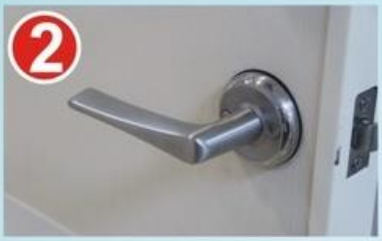

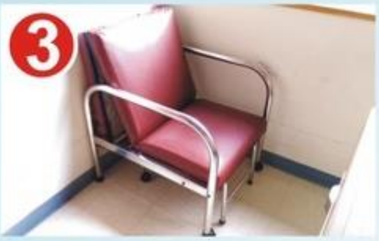

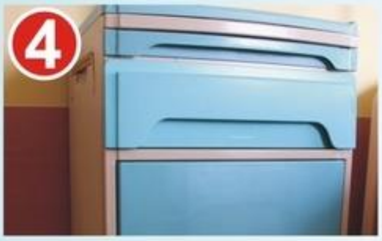

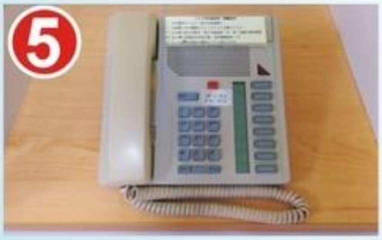

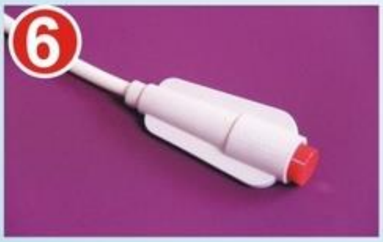

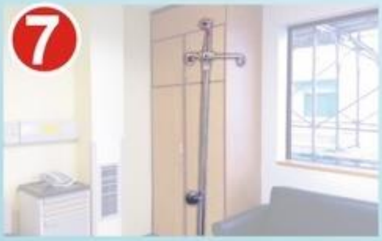

7*H27CM

DATE

/ /

Room No. of First terminal Cleaning

1 light switch	8 bed rail
2 door knob	9 control panel
3 chair	10 overbed table
4 table handle	11 monitor panel
5 telephone	12 ECG lead machine
6 nursing call	13 suction controller
7 IV pole	14 toilet seat/ handle

Terminal cleaning record

Signature

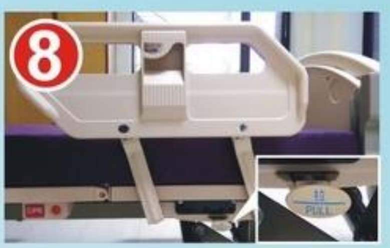

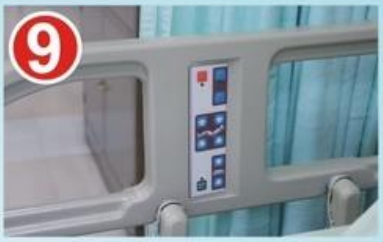

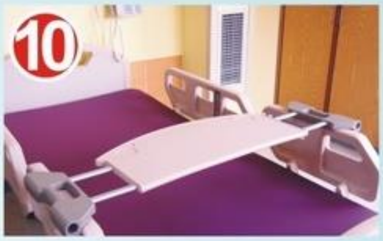

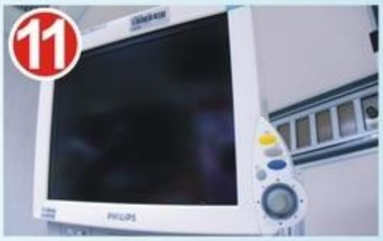

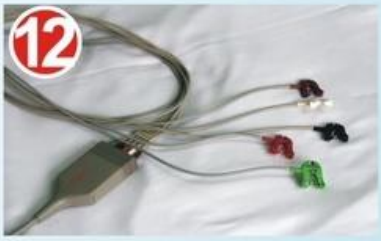

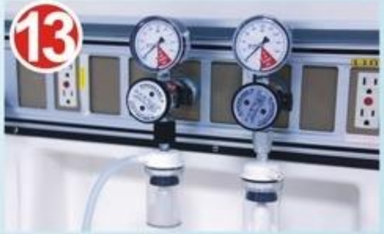

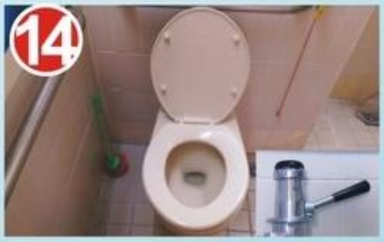

Checklist for Terminal Cleaning

210*H305MM
